# Pathophysiological Mechanisms of Chronic Venous Disease and Implications for Venoactive Drug Therapy

**DOI:** 10.3390/ijms19061669

**Published:** 2018-06-05

**Authors:** Armando Mansilha, Joel Sousa

**Affiliations:** 1 Departamento de Cirurgia e Fisiologia, Faculdade de Medicina da Universidade do Porto, 4200-319 Porto, Portugal; joel.sousa@jmellosaude.pt; 2 Departamento de Angiologia e Cirurgia Vascular, Hospital CUF Porto, 4100-180 Porto, Portugal

**Keywords:** chronic venous disease, chronic venous insufficiency, pathophysiology, inflammation, endothelial dysfunction, venoactive drugs, flavonoid, micronized purified flavonoid fraction, MPFF

## Abstract

Chronic venous disease (CVD) is a common pathology, with significant physical and psychological impacts for patients and high economic costs for national healthcare systems. Throughout the last decades, several risk factors for this condition have been identified, but only recently, have the roles of inflammation and endothelial dysfunction been properly assessed. Although still incompletely understood, current knowledge of the pathophysiological mechanisms of CVD reveals several potential targets and strategies for therapeutic intervention, some of which are addressable by currently available venoactive drugs. The roles of these drugs in the clinical improvement of venous tone and contractility, reduction of edema and inflammation, as well as in improved microcirculation and venous ulcer healing have been studied extensively, with favorable results reported in the literature. Here, we aim to review these pathophysiological mechanisms and their implications regarding currently available venoactive drug therapies.

## 1. Introduction

Chronic venous disease (CVD) is a common pathology of the circulatory system, representing both a significant medical problem for patients and a substantial burden for healthcare systems [1,2]. This condition encompasses a cascade of pathophysiological consequences arising from venous hypertension in the lower extremities, which can have variable etiologies. Most commonly, this venous hypertension is associated with venous reflux due to poorly functioning or incompetent venous valves, which ultimately reduces venous return, leading to blood pooling, hypoxia and inflammation.

The signs of CVD in the legs are variable, and include telangiectasia (spider veins), varicose veins, edema or skin changes (eczema, hyperpigmentation, and induration). In more severe cases, skin or venous ulceration can also be present.

Symptoms include various degrees and forms of leg discomfort, such as pain or aching, swelling, heaviness, cramps, and burning, all of which can significantly impact quality of life and lead to lost workdays. The spectrum of CVD clinical presentations has been defined according to the Clinical, Etiological, Anatomical, and Pathophysiological (CEAP) classification system, for which the clinical description ranges from C0 to C6 [3] (Table 1). At the earliest clinical stages (class C0), abnormal or reduced venous flow may be present but is without signs or symptoms. The term “chronic venous insufficiency” (CVI) is used when more advanced signs of CVD (classes C3−C6) are present. The etiologic, anatomic, and pathophysiologic descriptions help to further define and specify the condition and may change over time.

Estimates for the prevalence of CVD in adults, typically diagnosed by the presence of varicose veins, have been reported to vary between 5% and 65%, depending on the population, and tend to be higher in Western countries than in developing countries [4,5]. However, the Vein Consult Program, an international, observational, prospective survey that included over 90,000 patients across different geographic regions, demonstrated that the prevalence of symptomatic CVD (C0s or higher) was roughly similar around the globe, with prevalences of 78% in Western Europe, 87% in Eastern Europe, 88% in Latin America, 85% in the Middle East, and 87% in the Far East [5].

Varicose veins and CVD are generally reported to be more common in women than in men [4,5,6,7]; a notion that has been reinforced by several cross-sectional epidemiological studies. CVD C1–C3 but not CVD/CVI (C4–C6) was more common in women in the Vein Consult Program [5], while in the Bonn Vein Study varicose veins, CVI, and leg symptoms were all more prevalent in women than in men [7]. In France, Carpentier et al. reported the prevalence of varicose veins to be 50.5% in women and 30.1% in men [6]. In the Edinburgh Vein Study published in 1999, the prevalence of varicose veins was initially found to be significantly higher in men (40%) than in women (32%) [8] although a follow-up report published in 2013 demonstrated that the incidences of varicose veins and CVI were in fact strongly associated with increasing age rather than with sex [9].

Indeed, established risk factors for CVD are increasing age and previous pregnancy [4,5,6,7,9,10,11,12]. For example, the prevalence of CVI increased in the Vein Consult Program from 22.7% in women 35−50 years of age to 36.4% in women over 65, and from 15.7% in men 35−50 years of age to 27.7% in men over 65 [5]. Previous pregnancy was associated with odds ratios (OR) of 1.98 for varicose veins [6] and 1.20 for venous reflux [13], whereas the risk of CVI was found to increase with the number of pregnancies [11]. Less consistent risk factors have been reported to be female sex alone (without previous pregnancy), family history of CVD, high body mass index, and occupations that involve prolonged standing or sitting [9,10,11,14].

Estimates for the incidence of CVD have been reported from cohorts with significant follow-up periods. The overall annual incidence of venous reflux was 0.9% in the Edinburgh Vein Study [15] and the annual incidence of varicose veins was reported to be 2.6% in women and 1.9% in men in the Framingham Study [16]. In a meta-analysis of patients with varicose veins, approximately 4% progressed to a higher clinical classification of CVD each year [17,18].

The symptoms of CVD can be a daily challenge for patients and can measurably decrease quality of life (QOL) [19,20]. Symptoms also limit the ability of patients to participate in social, physical, or occupational activities, with financial consequences due to lost work time or disability [21,22]. In patients with symptomatic CVD, QOL decreases with increasing symptoms, severity, and CEAP class [19]. Among the scales used to measure QOL in CVD patients, the ChronIc Venous dIsease quality of life Questionnaires (CIVIQ)-20 and CIVIQ-14 have been validated in multiple countries and have been shown to be sensitive indicators of changes in CVD symptoms [23,24]. Parameters in the CIVIQ-20 and CIVIQ-14 are physical, psychological, social, and pain, which can be combined to yield a global score between 0 (worst) and 100 (best) [25]. Such scales provide a sensitive means of measuring disease progression, as well as clinical improvement in response to treatment and the corresponding increases in QOL [23,26]. Also, variations in QOL may be more sensitive than changes in CEAP class and are, therefore, potentially more useful for patient follow-up [19].

CVD also represents a substantial burden for healthcare systems. Costs accrue from inpatient and outpatient hospital care, treatments, and losses in productivity and work days due to disability, especially in cases with venous ulceration [27,28,29]. Total annual healthcare costs for venous and lymphatic disorders have been estimated to be €2.18 billion for Germany in 2006 and €2.24 billion for France in 1991 [21,30]. Da Silva et al. reported that, in Portugal, patients with venous ulcers required an average of 32 dressings and approximately 40 days of disability, with 12.5% taking early retirement [27]. The total healthcare system costs of CVD have been estimated at 1−2% of the healthcare budgets in western European countries and the USA, with 22% of nurses’ time spent on the treatment of leg ulcers [21,28,30].

With the increasing age of the global population, coupled with the rising prevalence of obesity as well as lifestyles and occupations that are progressively more sedentary, the prevalence of CVD, venous ulceration, and its associated burdens are expected to increase. These trends signal an urgent demand to better understand CVD and develop treatments targeting its pathophysiological mechanisms.

## 2. Pathophysiology of Chronic Venous Disease (CVD)

Individuals with venous hypertension, which may occur consequent to predisposing factors such as advanced age, obesity, previous pregnancy, family history, and/or environmental or occupational factors are at high risk of developing CVD [2,6,10,11,14]. In most cases, this hypertension is associated with poorly functioning venous valves and venous reflux, though it may also result from previous venous obstruction.

In normal healthy individuals, blood is pumped out the legs by calf muscle action [31]. Ineffective calf muscle pumping due to leg immobility from prolonged sitting or standing, inactivity, or obesity contributes to venous blood pooling. The subsequent venous hypertension in the lower extremities initiates a circular loop of vascular and inflammatory phenomena that potentiate the hypertension [32].

**Figure 1:**
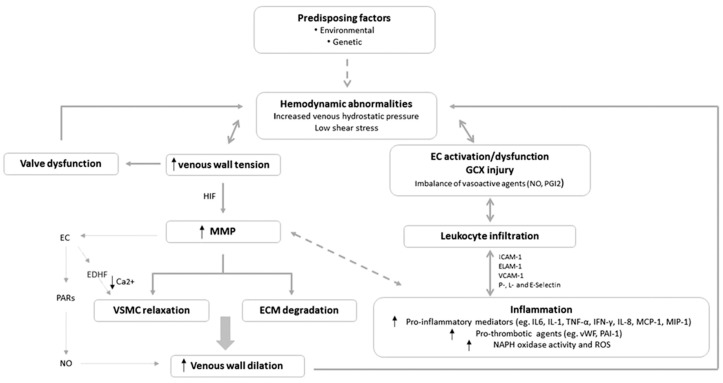
Schematic representation of the interplay of the pathophysiological mechanisms contributing to CVD development. Predisposing factors such as female sex, pregnancy, family history, obesity, sedentary lifestyles and occupations can lead to hemodynamic abnormalities in the veins of the lower legs, which may or may not be preceded by venal valve dysfunction. Elevated venous hydrostatic pressure and low shear stress lead to increased venous wall tension and distension, followed by activation of matrix metalloproteinases (MMP) through upregulation mediated by the hypoxia inducible factor (HIF) transcription factors. MMPs contribute to: degradation of the extracellular matrix (ECM); vascular smooth muscle cell (VSMC) relaxation through release of endothelium-derived hyperpolarizing factor (EDHF) and inhibition of calcium mobilization; and further venous wall dilatation. Inappropriate endothelial cell (EC) activation and injury to the glycocalyx (GCX) result in leukocyte infiltration and activation, setting up a proinflammatory environment within the vein wall, which in turn leads to further vein wall deterioration, leakage, tissue inflammation, and local prothrombotic abnormalities. Abbreviations: ELAM-1, endothelial-leukocyte adhesion molecule-1; ICAM-1, intercellular adhesion molecule-1; IL-, interleukin-; IFN-γ, interferon gamma; MCP-1, monocyte chemoattractant protein 1; MIP-1, macrophage inflammatory protein-1; NO, nitic oxide; PARs, protease-activated receptors; PGI2, prostaglandin I2/prostacyclin; ROS, reactive oxygen species; TNF-α, tumor necrosis factor alpha; VCAM-1, vascular cell adhesion molecule-1. (Reproduced from [34] with permission from the publisher).

There is increasing evidence to support the notion that among several possible trigger mechanisms, CVD is primarily a blood pressure-driven inflammatory disease, although the sequence of events is not fully understood and may differ depending on the risk factors involved. Elevated venous pressure and a shift in fluid shear stress generate an abnormal biomechanical environment in veins, vein walls, and valves [33]. These hemodynamic and biomechanical abnormalities may induce endothelium dysfunction, leading to early release and activation of enzymes that degrade the extracellular matrix and, in turn, set in motion a cascade of leukocyte infiltration and inflammation [33] (Figure 1).

As a consequence of this endothelial cell activation, several growth factors are secreted, inducing hypertrophic effects in vascular smooth muscle cells (VSMCs), endothelial cells, and extracellular matrix. Vascular endothelial growth factor (VEGF) [35,36], platelet-derived growth factor [37], angiotensin 2, endothelin 1, and fibroblast growth factor β (FGF-β) [38,39] are some examples of the growth factors found to be elevated in varicose veins, all of which contribute to VSMC proliferation, which in turn disrupts the normal structure of the vein wall [1].

Since VSMC must dedifferentiate to undergo proliferation [40], proliferating VSMC have limited functionality and contractility and are therefore poorly suited for maintaining vessel tone in the context of chronic high pressure. Imbalanced collagen synthesis by VSMCs has been implicated in the altered elasticity and increased rigidity of varicose veins, which weakens the vein wall and renders it incapable of maintaining shape and integrity against high pressure [40,41]. Elevated concentrations of transforming growth factor β1 (TGF-β1) also contribute to the disruption of extracellular matrix homeostasis, as TGF-β1 stimulates release of tissue inhibitors of the matrix metalloproteinases (MMPs) responsible for degrading extracellular matrix proteins [34].

Under conditions of normal blood flow and high shear stress, leukocytes remain unattached or roll along the lumen of the vein by adopting a rounded shape without pseudopods and by producing low levels of cell surface adhesion molecules [42,43]. Additionally, venous endothelial cells respond to pulsatile laminar shear stress by producing antithrombotic and anti-inflammatory factors, such as nitric oxide and prostacyclin, and by limiting the proinflammatory action of tumor necrosis factor (TNF-α) [44]. When venous reflux and altered hemodynamics with elevated pressure are present, reduced and abnormal fluid shear stresses are produced in the vein wall, disrupting normal venous homeostasis and activating the production of inflammatory factors by endothelial cells, such as monocyte chemoattractant protein 1 (MCP-1) and bone morphogenic protein 4 [32,33,45]. Endothelial cells also increase production of cell adhesion molecules, such as intercellular adhesion molecule 1 (ICAM-1) and vascular cell adhesion molecule 1 (VCAM-1), which promote leukocyte adhesion and migration [33].

A protective layer of glycoproteins and extracellular matrix components called the glycocalyx, overlies the endothelial cell layer and is a critical component of the vascular lumen important for mechanotransduction of shear stress and vein integrity [46]. Damage to the glycocalyx may occur through chronic distention due to hypertension, degradation due to low shear stress, or enzymatic cleavage by MMPs may trigger prothrombotic processes, as well as increased permeability and leukocyte adhesion [47,48]. All these features combine in CVD to produce a persistent proinflammatory and prothrombotic environment that leads to leukocyte activation, attachment, and transmigration, as well as secretion of other proinflammatory cytokines. Many studies confirm the role of inflammation in CVD, reporting increased expression of inflammatory markers, in vitro, in pre-clinical studies, and in patients with CVD [37,49,50]. Vascular endothelial cell cultures obtained from varicose veins were found to exhibit high levels of pro-inflammatory surface markers (CD31, CD146, and ICAM-1) and cytokines (osteoprotegerin and VEGF) [51]. Sola et al. reported distinct chemokine expression patterns in varicose veins due to significant up-regulation of MCP-1 and interleukin (IL)-8/CXCL8 and elevated expression of interferon-γ-inducible protein-10/CXCL10), RANTES/CCL5, macrophage-inflammatory protein-1a/CCL and -1b/CCL4 mRNA [50]. Poredos et al. demonstrated that the circulating inflammatory markers high sensitivity C-reactive protein and IL-6, as well as the fibrin degradation product, D-dimer, and von Willebrand factor were significantly higher in the blood samples taken from varicose veins, compared to those from systemic blood [49].

The idea that venous leukocyte activation and adhesion is increased in CVD is consistent with observations of enhanced leukocyte trapping in the lower extremities of CVD patients. Even in healthy persons, blood returning from the feet after an extended period of inactivity contains 15–20% fewer leukocytes than the arterial blood entering the feet [52]. However, this trapping effect is more pronounced in CVD patients, whose leukocyte counts in returning venous blood were 24% lower than in normal subjects [53]. In addition, immunohistochemical analysis of skin biopsies from the affected tissues of CVD patients revealed elevated numbers of leukocytes, especially T lymphocytes and macrophages [54]. Persistent accumulation of leukocytes in the legs, many of which migrate through the activated endothelium and the distended and damaged walls of the small veins and post-capillary venules into the surrounding tissue, is believed to be the basis for the chronic inflammation and lipodermatosclerotic skin changes associated with advanced CVD.

The altered hemodynamics and reduced venous return in the large veins of the legs result in the shunting of blood and fluid to smaller veins and capillaries of the skin, where pressure is also increased [55]. As a consequence, capillary beds and microvasculature become chronically dilated, dense, elongated, and tortuous [56]. Damage to the glycocalyx and endothelium in these abnormal pressurized capillaries leads to increased permeability, edema, leakage of red blood cells, and leukocyte infiltration and activation. Elevated levels of MMPs in the chronically inflamed dermal tissues contribute to excessive breakdown of extracellular matrix and collagen, which can lead to impaired healing and ulceration [57,58]. Abnormal capillary remodeling and permeability has also been linked to the high plasma levels of vascular endothelial growth factor found in CVD patients, especially those with skin changes [36,59]. Development of dermal tissue fibrosis may be due to the high levels of TGF-β1 present in the skin of the lower legs of CVD patients [60] because TGF-β1, produced by activated leukocytes, stimulates production of excess fibrinogen and collagen, which leads to fibrosis. Finally, the breakdown of extravasated red cells and the subsequent release of hemoglobin and ferric iron into the surrounding structures, increase the oxidative state of the tissue, which increases MMP activity, exacerbates tissue damage, and further impairs wound healing [61].

## 3. Implications for Treatment

Although still incompletely understood, the current knowledge of the pathophysiological mechanisms of CVD reveals many potential targets and strategies for therapeutic intervention. Early prevention of venous hypertension in the lower extremities is, in theory, the most effective way to prevent CVD. Nonetheless, in its earlier stages, venous hypertension is essentially asymptomatic and patients generally do not seek treatment until the symptoms of CVD appear. Hence, proper identification of individuals with one or more risk factors for venous hypertension and CVD could help identify those warranting early intervention. In fact, given the high societal costs of CVD and venous ulcer treatment, as well as the high burdens that CVD poses on patients and healthcare systems, screening programs aiming to identify and treat patients with asymptomatic venous hypertension, or those at risk of it, could reduce both the prevalence of CVD, as well as treatment costs associated with it.

In patients presenting with varicose veins or early stage CVD, conservative treatment with compression stockings is generally the standard treatment option used to reduce symptoms and prevent disease progression [2]. Although effective, with general improvement of all stages of CVD, as well as ulcer healing, compression stockings have poor treatment compliance, which limits their effectiveness. For that reason, many CVD patients warrant pharmacological treatment. Fortunately, several phlebotonics, or venoactive drugs, are currently available that intervene with many of the pathophysiological mechanisms of CVD (Table 2). These drugs offer promising pharmacological efficacy and safety profiles, and most are derived from natural flavonoids extracted from plants [62].

A recent Cochrane systematic review reported a meta-analysis of randomized, double-blind, placebo-controlled clinical trials investigating the efficacy of several venoactive drugs (such as rutosides, hidrosmin, diosmin, and calcium dobesilate) in the treatment of CVD [62]. The results indicated that, overall, venoactive drugs have significant beneficial effects on edema and may have benefits on some other signs and symptoms of CVD compared to placebo. However, objective outcomes were limited and more studies are needed to establish the efficacy of these treatments for other clinically important outcomes.

### 3.1. Micronized Purified Flavonoid Fraction (MPFF/Daflon^®^)

Micronized purified flavonoid fraction (MPFF; e.g., Daflon^®^) consists of 90% diosmin and 10% other active concomitant flavonoids (hesperidin, diosmetin, linarin, and isorhoifolin) and is currently one of the most widely available and prescribed venoactive drugs, and the most well-studied [63]. Diosmin is synthesized from hesperidin, which is extracted from a particular type of small immature orange, and the mixture is micronized to particles of <2-μm diameter to improve bioavailability.

The effects of MPFF have been demonstrated in both clinical and non-clinical studies, with reported improvements in venous tone and contractility, microcirculation, trophic disorders, and venous ulcer healing, and reductions in edema, inflammation, leukocyte adhesion and activation, and inflammatory mediator production [64]. A recent meta-analysis of randomized, double-blind, placebo-controlled clinical trials investigating the effectiveness of MPFF treatment in improving the symptoms, signs, and QOL in CVD patients identified 7 studies involving 1692 patients [65]. Most of the evidence was high quality and indicated that MPFF was highly effective at improving symptoms of leg pain, heaviness, and feeling of swelling (risk ratios (RR) of 0.35−0.53, *p* < 0.0001); cramps (RR 0.51, *p* = 0.02); paresthesia (RR 0.45, *p* = 0.03); skin changes (RR 0.18, *p* < 0.001); and burning sensation (standard mean difference (SMD) −0.46, 95% CI −0.78 to −0.14), as well as the signs of ankle swelling/circumference (SMD −0.59, 95% CI −1.15 to −0.02). QOL also improved (SMD −0.21, 95% CI −0.37 to −0.04).

#### 3.1.1. Improved Venous Tone and Contractility

Clinical and non-clinical studies support the notion that MPFF generally improves venous tone and contractility. Non-clinical studies in isolated rat veins found that diosmin directly enhanced sympathetic-mediated venous contractility and increased calcium sensitivity and contractility [66,67]. In similar studies in varicose human saphenous veins, the reported mechanisms of action were different although with similar results, since diosmin potentiated dose-dependent norepinephrine-induced contractility [68].

Regarding the clinical efficacy of MPFF in improving venous tone, two controlled clinical trials are particularly important. Barbe et al., in a study of women with various grades of CVD, demonstrated that MPFF treatment was associated with improvements in venous distension, capacitance, and tone [69]. Ibebguna et al. later demonstrated that, in women with abnormal venous elasticity and a high risk of developing varicose veins, MPFF treatment for 4 weeks significantly improved venous tone, as measured by elastic K modulus, from 10,200 ± 3900 N/m^2^ to 14,200 ± 5100 N/m^2^ (*p* < 0.02) [70].

#### 3.1.2. Reduction of Edema, Inflammation, Leukocyte Adhesion and Activation, Valve Deterioration, and Production of Inflammatory Mediators

**Figure 2:**
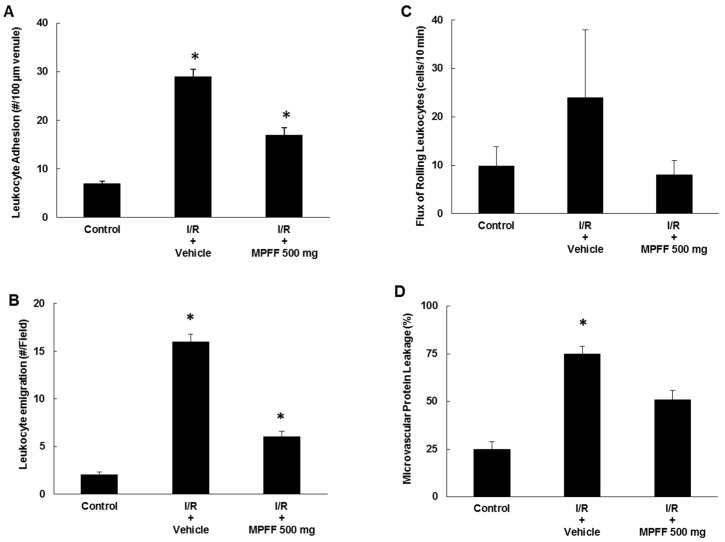
MPFF 500 mg attenuates the postischemic increases in leukocyte adhesion (**A**) and emigration (**B**), flux of rolling leukocytes (**C**), and microvascular protein leakage (**D**) in rat cremaster muscles. I/R = ischemia/reperfusion. Statistically different from * control (nonischemic conditions) and ^+^ vehicle: *p* < 0.05. (Reproduced from [73] with permission from the publisher).

In addition to its venotonic effects, MPFF has demonstrated various anti-inflammatory properties. In several animal models, MPFF treatment was shown to reduce leukocyte adhesion to vascular endothelium. In a hamster ischemia-reperfusion model, neutrophil adhesion in post-capillary venules was lower in animals pre-treated with MPFF than in control animals [71,72]. Similar findings were obtained in two rat ischemia-reperfusion models employing the cremaster muscle and mesentery vein [73] (Figure 2).

One mechanism by which MPFF may prevent leukocyte adhesion to damaged epithelium is by inhibiting the production of the surface molecules that mediate adhesion and activation. In CVD patients, MPFF was found to selectively reduce the expression of L-selectin/CD62-L on monocytes and neutrophils after treatment for 60 days [59]. In a rat model of chronic venous hypertension initiated by local venous occlusion followed by reperfusion (which also results in leukocyte adhesion and activation), MPFF treatment significantly mitigated these inflammatory processes and CD62-L expression in neutrophils in a dose-dependent manner [74]. In another rat model of venous hypertension and venous wall inflammation caused by femoral arterial-venous fistula, MPFF treatment significantly delayed the development of venous reflux and valve leaflet shortening in response to the elevated pressure, mitigated reductions in valve leaflet width and height, and decreased granulocyte and macrophage infiltration [75,76]. In a novel and recent model of chronic venous hypertension and disease in the hamster, iliac vein ligature induced a progressive increase in hind limb venous pressure over a period of 6–10 weeks that resulted in increased numbers of rolling and adherent lymphocytes, decreased functional capillary density, and enlarged venules [77]. In this model, MPFF treatment twice daily for 8 weeks significantly and effectively prevented these pathological consequences and was superior to either diosmin or the active concomitant flavonoid combination alone, suggesting a synergistic effect [77].

MPFF has been shown to reduce inflammatory marker concentrations locally and in the circulation. MPFF treatment for 2 weeks prior to sclerotherapy and 2 months after the procedure in patients with mild CVD (C1) resulted in lower levels of histamine, C-reactive protein, IL-1, TNF-α, and VEGF in blood taken from treated veins [78]. MPFF treatment for 12 weeks was also associated with significant reductions in systemic blood concentrations of endothelin-1 and TNF-α and with significantly increased antioxidant enzyme ratios in women with CVD [79]. This latter effect is particularly important since excess oxidative stress (low antioxidant enzyme ratios) leads to excessive production of radical oxygen species and has been reported in CVD patients [80]. This may contribute to leukocyte activation, inflammation, and vein wall injury, whereas higher antioxidant enzyme ratios may have protective effects.

**Figure 3:**
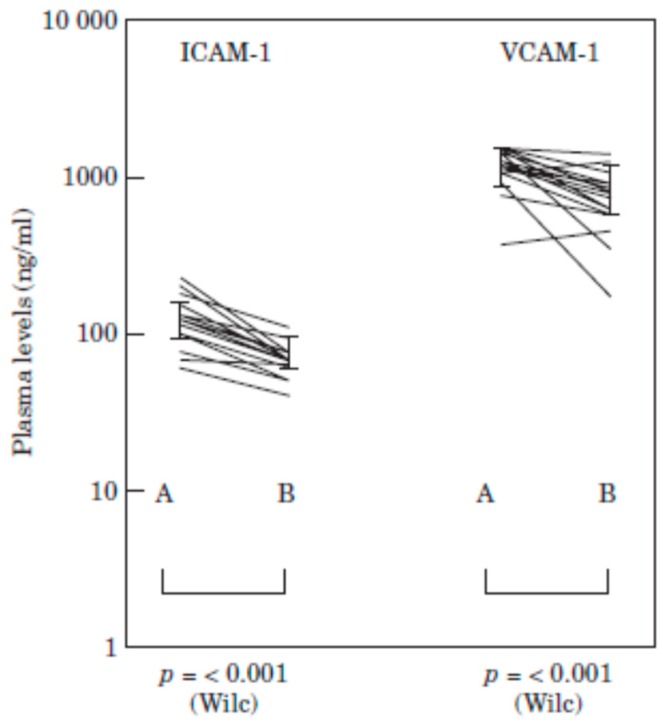
Changes in plasma levels if ICAM-1 and VCAM-1 in patients with CVD before (A) and after (B) 60 days of treatment with MPFF. A logarithmic scale has been used because of the differences in the absolute levels of the two molecules. *p*-levels are Wilcoxon ranked-sum test. The thick lines join the respective median levels. Vertical lines represent interquartile range. (Reproduced from [81] with permission from the publisher).

Coupled with its inhibitory effects on cells of the immune system, MPFF treatment may also reduce endothelial cell activity in CVD, which is an important trigger of inflammation and thrombosis. In patients with C2 to C5 CVD, MPFF treatment for 60 days reduced plasma ICAM-1 concentrations by 32% and plasma VCAM concentrations by 29% [81] (Figure 3). In another study in CVD patients with skin changes (C4), MPFF treatment for 60 days significantly reduced plasma VEGF concentrations by 42% (*p* < 0.02) [82]. These findings suggest that MPFF may help maintain endothelium quiescence in veins, preventing the binding and activation of leukocytes through these cell adhesion molecules. In addition, subsequent hypertrophic responses mediated by VEGF may also be reduced.

MPFF has also demonstrated clinically beneficial effects on leg edema. In a randomized controlled clinical trial in 200 CVD patients, MPFF treatment for 2 months significantly (*p* < 0.001) reduced ankle circumference [83], whereas in another trial in patients with leg edema associated with varicose veins, 6 weeks of MPFF treatment significantly reduced leg volume by 392 mL (12%; *p* < 0.001) [84]. MPFF treatment was also found to significantly reduce ankle edema compared to placebo in a meta-analysis of studies encompassing 463 CVD patients [85] and to reduce evening great saphenous vein diameters in patients with transient venous reflux due to daily orthostatic loading [86,87]. In patients with cyclic edema, MPFF treatment for 6 weeks significantly improved capillary hyperpermeability (*p* = 0.02) compared to the placebo treatment, and this improvement was accompanied by significant weight loss (*p* < 0.05) and a significant reduction (*p* < 0.05) in the sensation of swelling [88]. After 3 months of MPFF treatment (500–2000 mg/day) in CVD patients, transcutaneous oxygen pressure significantly increased (*p* < 0.001), transcutaneous carbon dioxide pressure significantly decreased (*p* < 0.001), and disease-related clinical symptoms (subjective manifestations and edema) were improved with no differences across the dosage groups [89].

The mechanisms behind the effects of MPFF on edema may be related to its demonstrated effects on reducing capillary leakage and improving capillary resistance. In patients with symptomatic capillary fragility, as evidenced by spontaneous bruising, frequent nosebleeds, petachiae, and conjunctival hemorrhage, MPFF treatment for 6 weeks significantly increased capillary resistance to rupture by negative suction cup pressure relative to placebo treatment [90]. In the hamster cheek pouch, immediate pretreatment with 10 or 30 mg/kg MPFF, or with equivalent doses of any of its constituent flavonoids, significantly reduced the number of leaky capillary sites produced by ischemia-reperfusion [91]. In a rabbit model of sclerotherapy, MPFF treatment for 21 days prior to initiation of inflammation prevented increases in venular diameter, preserved functional capillary density, and reduced capillary leakage (*p* < 0.001) [92].

#### 3.1.3. Improvements in Microcirculation, Trophic Disorders, and Venous Ulcer Healing

MPFF may also have beneficial effects on ulcer healing, but this outcome has not been extensively investigated in clinical trials. Guilhou et al., in a randomized, double-blind, placebo-controlled study in 105 CVD patients with venous ulcers who also wore compression stockings, demonstrated that complete ulcer healing rates for all patients were 26.5% in the MPFF treatment group (2 months) and 11.5% in the placebo group, although no differences were observed in the risk ratio of not healing (RR 0.83, 95% CI 0.69–1.0) [93]. However, in patients with smaller ulcers (≤10 cm in diameter), healing rates were also higher in the MPFF treatment group (31.8% vs. 12.8%) and the risk ratio of not healing (RR 0.78, 95% CI 0.62–0.98) was statistically significant. Two non-blinded similar studies compared ulcer healing rates in CVD patients receiving compression treatment with MPFF for 6 months versus patients receiving compression treatment alone [94,95]. After 6 months, ulcer healing rates were 46.5% in treated patients vs. 27.5% in untreated patients in one study and 64.6% vs. 41.2%, respectively, in the other study. Together, these findings suggest that MPFF improves ulcer healing with compression therapy, but additional clinical investigation is needed.

The effects of MPFF treatment on skin trophic disorders have been investigated in several randomized controlled clinical trials, although the reported evidence is conflicting. In two studies encompassing a total of 75 CVD patients treated with MPFF, skin trophic improvement was not significantly better than in patients treated with placebo [96,97]. On the other hand, in two larger trials in 160 and 200 CVD patients, skin trophic changes persisted in significantly fewer patients treated with MPFF (82.5% and 86%, respectively) than in those treated with placebo (95.0% and 96%, respectively) [83,98]. In a pooled analysis of all four studies, the RR of 0.87 (95% CI 0.81–0.94) for persistence of the skin trophic disorder with MPFF treatment vs. placebo, indicated a statistically significant benefit with MPFF [62].

Along with the efficacy of MPFF treatment in several objective clinical outcomes, CVD symptoms and QOL have also been reported to improve with MPFF treatment. Compared to placebo, MPFF treatment was associated with statistically significant improvements in functional discomfort, heaviness, pain, nocturnal cramps, and the sensations of swelling and heat [98]. A pooled analysis of additional studies that assessed CVD symptoms found statistically significant benefits for MPFF treatment in reducing symptoms of cramping, lower leg heaviness, and lower leg swelling, but not for pain or itching [62]. In a study in 5052 symptomatic CVD patients (C0–C4) who received MPFF treatment for 6 months, CIVIQ-20 QOL scores demonstrated significant and continuous improvement in QOL [99], whereas in a randomized controlled clinical trial in 592 CVD patients (C3–C4), MPFF treatment was associated with a significantly greater improvement of 3.1% in the CIVIQ-20 score (*p* = 0.040) [100].

### 3.2. Rutosides

Rutosides or rutins represent another class of venoactive bioflavonoids and have been reported to have anti-inflammatory properties and to improve CVD signs and symptoms. Rutoside (as pentahydroxyflavone glycoside) was found to be a potent inhibitor of inflammation-related gene expression in activated human macrophages cultured in vitro, and to inhibit the release of nitric oxide, TNF-α, IL-1 and IL-6 from these cells [101]. In adjuvant-induced arthritic rats, rutoside reduced the clinical signs of arthritis, which correlated with the inhibition of inflammatory cytokine production measured in rat sera and in the human macrophages [101]. Rutin was also found to significantly inhibit nitric oxide and TNF-α production, as well as myeloperoxidase activity in human peripheral blood neutrophils [102]. In several clinical studies in CVD patients, rutoside preparations were reported to significantly reduce edema [103,104,105] and leg volume [106], and reduced severity of lower leg pain, leg cramps, heaviness, and itching [103,106,107]. In a pooled analysis of randomized controlled clinical trials in CVD patients, these venoactive compounds were found to have significant benefits for edema, leg volume, lower leg pain, and leg heaviness [62].

### 3.3. Calcium Dobesilate

Calcium dobesilate (2,5-dihydroxy-benzenesulfonate) is a synthetic drug with vasoprotective and antithrombotic properties that has been widely used in the treatment of diabetic retinopathy and CVD. It may act at the vascular endothelium, as it has been shown to reduce capillary hyperpermeability associated with diabetes mellitus [108], inhibit platelet aggregation [109], and reduce whole blood viscosity [110]. Consistent with these effects, rat endothelial cell preparations incubated with calcium dobesilate in vitro exhibited enhanced nitric oxide-synthase activity, which is important for vascular homeostasis [111]. Calcium dobesilate was also shown to inhibit microsomal prostaglandin synthesis in vitro, and to reduce the viscoelasticity of whole blood and plasma in patients with ischemic disease after 14 days of treatment [112]. These actions are likely the basis for edema-reducing effects observed in CVD patients treated with calcium dobesilate. In three studies that measured lower leg volume in CVD patients following calcium dobesilate treatment for 4−8 weeks, leg edema was significantly reduced compared to patients treated with placebo [113,114,115]. Pooled analysis of these clinical results revealed that calcium dobesilate treatment was associated with significant improvements in objective measurements of leg edema and in subjective symptoms of leg pain, restless legs, and leg swelling [62]. However, in a recent well-designed randomized, parallel, double-blind, placebo-controlled clinical trial in over 500 CVD patients, calcium dobesilate treatment for 3 months elicited no significant improvements compared to placebo in leg edema, CVD symptoms, or QOL [116]. Taken together, these results suggest that calcium dobesilate may have beneficial effects in CVD but that further studies are needed to establish a definitive role for this treatment.

### 3.4. Sulodexide

As integrity of the glycocalyx is critical for maintaining vein homeostasis, treatments that prevent glycocalyx damage or breakdown and/or promote its repair could help protect the endothelium and reduce the inflammatory cascades stemming from the activation of endothelial cells [34,46]. Sulodexide is a highly purified glycosaminoglycan mixture, consisting of low molecular weight heparin (80%) and dermatan sulfate (20%), both of which are components of the glycoproteins that form the glycocalyx [117]. In patients with type 2 diabetes, which is commonly associated with disrupted glycocalyx and elevated vascular permeability, sulodexide treatment for 2 months was associated with partial restoration of glycocalyx thickness toward control values and by a reduction in the transcapillary permeability to albumin [118]. Sulodexide has also been shown to have anti-inflammatory and anti-apoptotic activities, to prevent leukocyte adhesion to endothelium, and to have a protective effect on the endothelial cell lining and vascular wall in microcirculation [119]. Specifically, in non-clinical and clinical studies, it lowered plasma concentrations of IL-1β and IL-8 in dialysis patients [120]; and inhibited release of MCP-1 and IL-6 and formation of free radicals in human umbilical vein endothelial cell cultures [121], neo-angiogenesis in a rat model of peritoneal perfusion [122], plasma TNF-α release in rat model of peritonitis [123], and release of a wide range of pro-inflammatory cytokines, chemokines, and colony stimulating factors from activated human macrophages [124]. More recently, sulodexide was shown to inhibit the release of IL-2, IL-12 (p70), IL-10 and VEGF from THP-1 monocytes stimulated in vitro with wound fluid collected from CVD patients with venous leg ulcers [125]. Sulodexide treatment may therefore help stem the proinflammatory cascades present in venous ulcers. Consistent with its protective effects on the vasculature, sulodexide significantly reduced secretion of pro- and complexed MMP-9 from cultured blood leukocytes and of MMP-1, MMP-9, and MMP-12 from THP-1 monocytes stimulated with wound fluid [126,127]. These results suggest additional mechanisms by which sulodexide treatment may prevent degradation of vascular extracellular matrix and collagen.

In CVD patients, sulodexide treatment was associated with reduced plasma concentrations of MMP-9, IL-6, and MCP-1 compared to pre-treatment concentrations [128]. In an uncontrolled study in 476 CVD patients that compared 60 days of treatment with different doses of sulodexide (50−100 mg/day), statistically significant and clinically relevant improvements in peripheral venous pressure were observed, along with significant reductions in other clinical signs and symptoms [129]. A recent open uncontrolled observational study in 450 CVD patients reported that 3 months of treatment with sulodexide significantly improved objective signs (erythema, skin temperature, induration) and all subjective symptoms of CVD (*p* < 0.0001) and significantly improved patient-assessed QOL scores in the CIVIQ questionnaire (*p* < 0.0001) [130].

Sulodexide treatment has also shown benefits in venous ulcer healing. In 235 patients with venous ulcers, those treated for 3 months with sulodexide exhibited significantly higher rates of complete ulcer healing and significantly greater reductions in ulcer surface area over time, compared to patients treated with placebo [131]. Similar benefits of this venotonic on ulcer healing in CVD patients have been reported for other studies [132], including one in which complete ulcer healing with local treatment was found to be significantly faster with a combined sulodexide plus MPFF treatment than with MPFF treatment alone [133]. A recent meta-analysis of three randomized controlled clinical trials investigating the effects of sulodexide on ulcer healing found that, although the quality of the evidence was low, the proportion of completely healed ulcers may be greater with local treatment (wound care and compression therapy) plus sulodexide treatment than with local treatment alone (49% vs. 30%; RR 1.66, 95% CI 1.30 to 2.12) [134]. Taken together, these findings suggest that sulodexide treatment may have clinical benefits for CVD signs and symptoms and for ulcer healing, though the recommendations for its use in the treatment of CVD could be strengthened by additional, well-controlled clinical trials.

## 4. Conclusions

CVD is a progressive medical condition that results from venous hypertension and can lead to diminished venous integrity and function, varicose veins, chronic inflammation, leg swelling, or even skin alterations and ulceration. It is already a major and growing global medical problem, with a high economic burden. Although widespread, this condition is particularly evident in Western countries, where risk factors for CVD are prevalent, sedentary lifestyles and occupations are common, and obesity is escalating. Because advanced age is the most critical risk factor, and the age of the population continues to increase, the prevalence of CVD, as well as the already substantial burdens of CVD-related morbidity, disability, and healthcare are all expected to increase in the coming decades. These alarming trends indicate an urgent need for treatments that can alleviate the symptoms of CVD and prevent its progression over time.

Chronic inflammation is the principal basis behind the pathophysiological mechanisms that potentiate disease progression and produce the signs and symptoms of CVD. Brought on by chronic venous hypertension, the accumulating leukocytes and damaged venous endothelium initiate inflammatory cascades in the distended veins, which then propagate into the microvasculature and surrounding tissues. This chronic inflammation further degrades venous integrity and function, resulting in diminished venous return, fluid accumulation, tissue fibrosis, atrophy, and ulceration in severe cases.

Venoactive drugs present pharmacological and clinical profiles that can be explained by their action at specific levels of CVD pathophysiology (Table 2). MPFF increases venous contractility in isolated vein preparations and increases venous tone in persons at risk for varicose veins [70], whereas these drugs as a class all exhibit broad anti-inflammatory activity. Several have been shown to reduce leukocyte adhesion and activation in different models of venous inflammation induced either by ischemia-reperfusion or by transient venous hypertension [71,73,75,76,77]. In humans, venoactive drugs reduce the concentration of plasma markers of inflammation (TNFα and other cytokines), endothelial activation (ICAM, VCAM), vascular hypertrophy and angiogenesis (VEGF), as well as the release of proteases involved in the breakdown of extracellular matrix and venous tissue remodeling. Most also appear to improve capillary resistance and decrease excess vascular permeability. The pharmacological actions of these drugs are the basis behind their demonstrated clinical benefits, including significant reductions in leg edema, skin trophic disorders, patient-reported symptoms, and ulcer healing time. Inappropriate endothelial activation in CVD is a mechanism common to other cardiovascular diseases and diabetes. Drugs that specifically target endothelial dysfunction in these diseases may also be beneficial in CVD and some such as angiotensin-converting enzyme inhibitors, statins, and agents that target endothelial NO synthase coupling may be worth investigating as adjunct therapies in future studies.

The pharmacological profiles and demonstrated clinical benefits of venoactive drugs provide the rationale behind their use in the treatment of CVD. As a class, these medications have excellent safety profiles, and most have been available for many years in Europe. MPFF and sulodexide are recommended in international guidelines for the treatment of CVD and venous ulcers but MPFF is the only one to obtain a grade 1B (based on a strong recommendation for use and moderate quality of evidence) in the relief of symptoms associated with C0s to C6 [135,136]. MPFF and sulodexide are also recommended in the clinical practice guidelines issued by the Society for Vascular Surgery and the American Venous Forum [137]. Further experience and controlled clinical investigation of venoactive drugs is likely to provide additional clinical evidence to better define the therapeutic benefits of these important treatments to CVD patients.

## Figures and Tables

**Table 1:** Clinical, Etiologic, Anatomic, and Pathophysiologic (CEAP) classifications of CVD.

Clinical Classification		Etiologic Classification		Anatomic Classification		Pathophysiologic Classification (Basic CEAP ^1^)	
C0	No visible or palpable signs of venous disease	Ec	Congenital	As	Superficial veins	Pr	Reflux
C0s	C0 with minor symptoms	Ep	Primary	Ap	Perforator veins	Po	Obstruction
C1	Telangiectasia or reticular veins	Es	Secondary (postthrombotic)	Ad	Deep veins	Pr,o	Reflux and obstruction
C2	Varicose veins	En	No venous cause identified	An	No venous location identified	Pn	No venous pathophysiology identifiable
C3	Edema						
C4a	Pigmentation and/or eczema						
C4b	Lipodermatosclerosis and/or atrophie blanche						
C5	Healed venous ulcer						
C6	Active venous ulcer						
S	Symptomatic, including ache, pain, tightness, skin irritation, heaviness, and muscle cramps, and other complaints attributable to venous dysfunction						
A	Asymptomatic						

^1^ Basic CEAP includes these descriptors only. In the Advanced CEAP classification, any of 18 named venous segments are added as locators for the venous pathology [3]. CVD, chronic venous disease.

**Table 2:** Pharmacological effects of venoactive drugs.

Venoactive Drug	Pharmacological Action	Clinical Benefit
MPFF/Daflon	Enhances sympathetic-mediated venous contractility and calcium sensitivity Reduces leukocyte adhesion; inhibits production of leukocyte adhesion molecules Mitigates venous valve deterioration and reflux Inhibits production of proinflammatory factors Increases antioxidant enzyme ratios Reduces endothelial cell activation; lowers serum concentrations of ICAM-1, VCAM, VEGF Increases capillary resistance, reduces capillary leakage	Improves venous tone Reduces leg edema Improves skin trophic disorders Improves ulcer healing Improves CVD symptoms and QOL
Rutosides	Potent inhibitor of inflammation-related gene expression Reduces production of inflammatory cytokines (NO, TNF-α, IL-1, IL-6) in macrophages and neutrophils	Reduces leg edema Improves CVD symptoms
Calcium dobesilate	May improve or maintain vascular endothelium function Reduces capillary hyperpermeability Inhibits platelet aggregation Reduces blood viscosity Increases NO-synthase activity Inhibits prostaglandin synthesis	Reduces leg edema Improves CVD symptoms
Sulodexide	Restores GCX integrity Reduces vascular and capillary permeability Protects vascular endothelium Anti-inflammatory, anti-apoptotic (IL-1β, IL-8, MCP-1, IL-6, TNF-α) Reduces secretion of MMP-9 from leukocytes	Reduces peripheral venous pressure Improves CVD symptoms and QOL Improves ulcer healing

Abbreviations: CVD, chronic venous disease; GCX, glycocalyx; ICAM, intercellular adhesion molecule; IL, interleukin; MCP, monocyte chemoattractant protein; MMP, matrix metalloproteinase; MPFF, micronized purified flavonoid fraction; NO, nitric oxide; QOL, quality of life; TNF, tumor necrosis factor; VCAM, vascular cell adhesion molecule; VEGF, vascular endothelial growth factor.
